# Influence of thickness and morphology of MoS_2_ on the performance of counter electrodes in dye-sensitized solar cells

**DOI:** 10.3762/bjnano.13.44

**Published:** 2022-06-17

**Authors:** Lam Thuy Thi Mai, Hai Viet Le, Ngan Kim Thi Nguyen, Van La Tran Pham, Thu Anh Thi Nguyen, Nguyen Thanh Le Huynh, Hoang Thai Nguyen

**Affiliations:** 1 Tra Vinh University, 126 Nguyen Thien Thanh Street, Ward 5, Tra Vinh City 940000, Vietnam; 2 University of Science, VNU-HCM, 227 Nguyen Van Cu Street, District 5, Ho Chi Minh City, 700000, Vietnam; 3 Vietnam National University Ho Chi Minh City, Linh Trung Ward, Thu Duc City, Ho Chi Minh City, 700000, Vietnam

**Keywords:** cyclic voltammetry (CV), dye-sensitized solar cells (DSSCs), electrocatalytic activity, honeycomb-like, molybdenum disulfide (MoS_2_), thin film

## Abstract

Non-platinum electrodes for photoelectric devices are challenging and attractive to the scientific community. A thin film of molybdenum disulfide (MoS_2_) was prepared on substrates coated with fluorine-doped tin oxide (FTO) to substitute the platinum counter electrode (CE) for dye-sensitized solar cells (DSSCs). Herein, we synthesized layered and honeycomb-like MoS_2_ thin films via the cyclic voltammetry (CV) route. Thickness and morphology of the MoS_2_ thin films were controlled via the concentration of precursor solution. The obtained results showed that MoS_2_ thin films formed at a low precursor concentration had a layered morphology while a honeycomb-like MoS_2_ thin film was formed at a high precursor concentration. Both types of MoS_2_ thin film were composed of 1T and 2H structures and exhibited excellent electrocatalytic activity for the I_3_^–/^I^−^ redox couple. DSSCs assembled using these MoS_2_ CEs showed a maximal power conversion efficiency of 7.33%. The short-circuit value reached 16.3 mA·cm^−2^, which was higher than that of a conventional Pt/FTO CE (15.3 mA·cm^−2^). This work reports for the first time the possibility to obtain a honeycomb-like MoS_2_ thin film morphology by the CV method and investigates the effect of film structure on the electrocatalytic activity and photovoltaic performance of CEs for DSSC application.

## Introduction

Since Grätzel’s first report in 1991, dye-sensitized solar cells (DSSCs) have been the subject of much research due to the easy fabrication process and respectable eﬃciency [1]. This promising third generation of solar cells contains a dye-adsorbed TiO_2_ photoanode, an iodide/triiodide electrolyte, and a platinum-based cathode, also known as the counter electrode (CE). However, the high cost of platinum has prevented the real-world application of DSSCs, which has led researchers to explore efficient cathode materials for DSSCs beyond platinum. To date, Pt replacement materials are divided into three categories, namely carbonaceous materials [2–5], conductive polymers [5], and transition metal compounds [6–8]. Transition metal compounds are considered a potential approach due to the high activity and acceptable price. Molybdenum disulfide (MoS_2_) has recently gained a lot of attention due to its layered structure, cost efficiency, and superior catalytic activity [9–16]. MoS_2_ exhibits layered structures with three types of crystal phase, that is, trigonal (1T), hexagonal (2H), and rhombohedral (3R). Considering electrocatalytic applications, the 1T metallic phase exhibits a higher catalytic activity than the 2H and 3R semiconductor phases [11,17]. Moreover, it is well known that the electrocatalytic activity of MoS_2_ strongly depends on the number of catalytically active sites located at the edge planes [1,18–19]. These unsaturated Mo and S edges of MoS_2_ enable the generation of the I_3_^–/^I^–^ redox couple, making it a potential CE for DSSCs. So far, MoS_2_-based CEs for DSSCs have been fabricated and investigated using various techniques such as chemical bath deposition [1], sputtering [2], hydrothermal synthesis [10–13], wet chemistry [14], thermal reduction [15], and electrodeposition (ED) [20]. Among these methods, ED shows many advances thank to its simplicity and rapidity. Additionally, it allows for the direct deposition of MoS_2_ thin films from liquid precursors onto various conducting substrates with easily controlled thickness and morphology.

Several reports have already been published that describe the control of structure and morphology of electrodeposited MoS_2_ to maximize its catalytic activity. Li et al. reported the synthesis of MoS_2_/graphene composite films on FTO, which were directly used as CE for DSSCs without further thermal treatment. The power conversion efficiency (PCE) of the DSSCs was 8.01%, which was comparable to that of a Pt CE (8.21%) [21]. Quy et al. prepared MoS_2_/FTO. The resulting DSSCs showed a PCE of 7.16%, similar to that of a Pt/FTO CE (7.48%). The MoS_2_ film was amorphous and contained agglomerated clusters of nanoparticles [22]. Recently, Gurulakshmi et al. reported on DSSCs using a flexible CE fabricated by electrodeposition of a MoS_2_ thin film onto a conductive FTO/PET substrate. The PCE of this flexible DSSCs reached 4.84%. The MoS_2_ film was composed of sheets with a length of about 6 µm and a thickness of about 500 nm [23]. Another report by Chang et al. mentioned the change in morphology of MoS_2_ from sphere-like shapes with large grain size to a uniform thin layer when changing the ED technique from potentiostatic (PS) mode to potential-reversal (PR) mode. This resulted in an improvement in PCE from 6.89% to 8.77% [24]. In general, above studies still have limits such as depositing MoS_2_ on graphene or carbon dots, instead of directly developing the FTO substrate. In addition, the effect of thickness and morphology of MoS_2_/FTO on the performance of DSSCs was not examined in these studies.

In this work, thin films of MoS_2_ with two different shapes (layered and honeycomb-like) were deposited on FTO substrates from an aqueous precursor solution containing (NH_4_)_6_Mo_7_O_24_·4H_2_O and Na_2_S by cyclic voltammetry (CV). Morphology and thickness of the MoS_2_ thin films were controlled by adjusting the concentration of the precursor solution. The electrochemical catalytic activity of the MoS_2_ thin films was investigated regarding the I_3_^–^/I^–^redox couple. The as-prepared MoS_2_ thin films were directly used as CE for DSSCs. The structure and morphology of the MoS_2_ thin films and their corresponding DSSC performance have been carefully evaluated. Furthermore, the effect of MoS_2_ film thickness on the performance of DSSCs has also been discussed. It should be noted that this is the first report dealing with the fabrication of MoS_2_ honeycomb-like thin films for DSSC application.

## Results and Discussion

### Electrodeposition of MoS_2_ thin films

**Figure 1:**
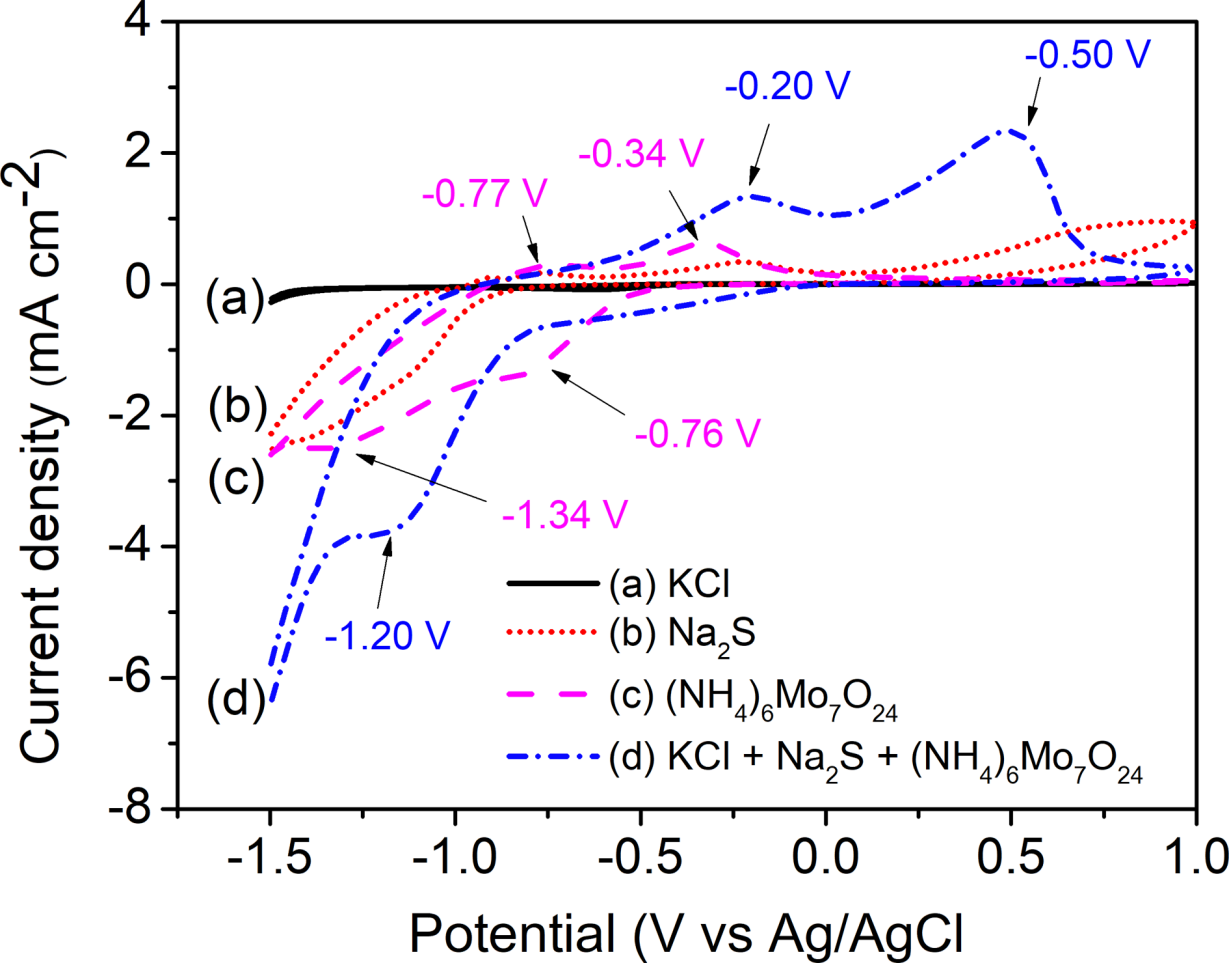
CV curves recorded in the solutions of (a) 0.1 M KCl, (b) 30 g/L Na_2_S, (c) 5 mM (NH_4_)_6_Mo_7_O_24_, and (d) a mixture of 30 g/L Na_2_S and 5 mM (NH_4_)_6_Mo_7_O_24_ in 0.1 M KCl, pH 6, using an FTO electrode, at scan rate of 100 mV·s^−1^.

Electrodeposition of MoS_2_ thin films was carried out from precursor solutions containing a mixture of (NH_4_)_6_Mo_7_O_24_ and Na_2_S in KCl electrolyte solution. To study the redox behavior of the solution, the CV curves for each component and the mixture solutions were recorded in the potential range from −1.5 V to 1.0 V (Figure 1). The blank KCl electrolyte exhibits a straight line around zero current, while the precursor solutions show redox peaks associated with the oxidation/reduction of the precursor ions on the surface of the FTO electrode. In detail, the CV recorded in Na_2_S solution shows a broad anodic peak around −0.50 V due to the oxidation of S^2−^ ions [25–26]. The CV curve of (NH_4_)_6_Mo_7_O_24_ solution exhibited two redox couple peaks at −0.34 V/−0.76 V and −0.77 V/−1.34 V attributed to the redox reactions of Mo_7_O_24_^6−^ and MoO_4_^2−^ ions, respectively [27]. The presence of MoO_4_^2−^ ions is due to the equilibrium in Equation 1, which occurs in acidic solution of (NH_4_)_6_Mo_7_O_24_ (the pH here is about 4.3). The CV recorded in the mixture solution showed two oxidation peaks at −0.20 V and −0.50 V attributed to the oxidation of Mo_7_O_24_^6−^ and S^2−^ ions, respectively. Moreover, a new reduction peak appeared around −1.20 V related to the reduction of MoS_4_^2−^ to form MoS_2_ as described in Equation 2. This CV behavior is similar to that of (NH_4_)_2_MoS_4_ reported by Falola and co-workers [28]. The formation of MoS_4_^2−^ ions in the mixture solution is detailed in Equations 1–4 [27–30]:


[3] $${\text{Na}}_{2}\text{S}\to 2{\text{Na}}^{+}+{\text{S}}^{2-},$$

[4] $${\left({\text{NH}}_{\text{4}}\right)}_{\text{6}}{\text{Mo}}_{\text{7}}{\text{O}}_{\text{24}}\cdot {\text{4H}}_{\text{2}}\text{O}\to {\text{Mo}}_{7}{\text{O}}_{24}{}^{6-}+6{\text{NH}}_{4}{}^{+}+4{\text{H}}_{2}\text{O},$$

[1]

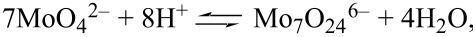




[5] $${\text{MoO}}_{4}{}^{2-}+6{\text{S}}^{2–}+12{\text{H}}^{+}\to {\text{MoS}}_{4}{}^{2-}+4{\text{H}}_{2},$$

[2] $${\text{MoS}}_{4}{}^{2-}+6{\text{H}}^{+}\text{+}2{\text{e}}^{-}\to {\text{MoS}}_{2}+2{\text{H}}_{2}\text{S}+{\text{H}}_{2}.$$

It should be noted that (NH_4_)_2_MoS_4_ is poorly soluble in water. Hence, the in situ synthesis of MoS_4_^2−^ from (NH_4_)_6_Mo_7_O_24_ (high solubility) and Na_2_S in acidic media (adjusted to pH 6) is very favorable to the preparation of MoS_4_^2−^ precursor solution. In this work, the optimal concentration ratio of (NH_4_)_2_Mo_7_O_24_ (mM) to Na_2_S (g/L) was found to be 1:6 (data not shown).

**Figure 2:**
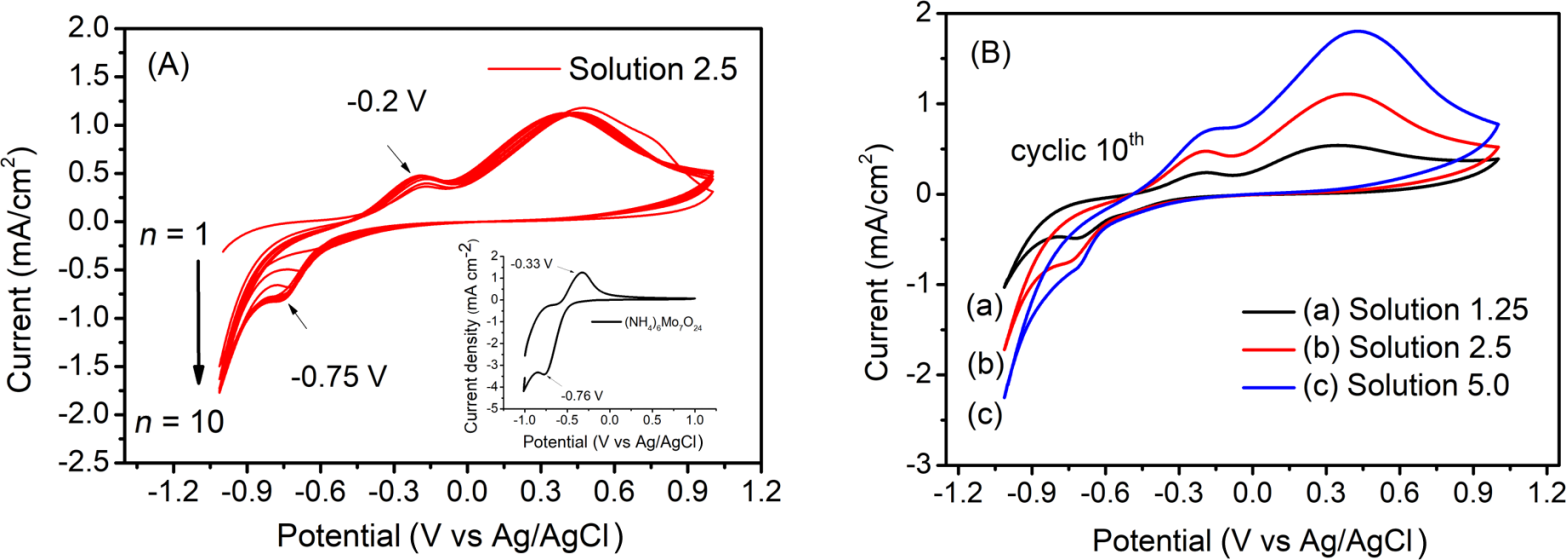
(a) CVs recorded during electrodeposition of MoS_2_ from solution 1.25; (b) comparison of the tenth cycle of CVs recorded in solutions 1.25, 2.5, and 5.0; a scan rate of 100 mV·s^−1^ was used.

It can be seen from the CV curve of the mixture solution that the reduction of MoS_4_^2−^ occurred beginning at a potential of −0.80 V. Electrodeposition of MoS_2_ at high overpotential leads to the formation of thick films [28]. To obtain thin films, we limited the deposition potential range of MoS_2_ to a range between −1.0 V and 1.0 V and studied the effect of the concentration of the precursor solution on the morphology and the electrocatalytic activity of the MoS_2_ thin films. The CVs (10 cycles) for the MoS_2_ electrodeposition from solution 2.5 (see Experimental section for the denomination of the sample solutions) are shown in Figure 2a. The comparison of the tenth cycle of the CV recorded in different concentrations of precursor solution (solution 1.25, 2.5, and 5.0) is shown in Figure 2b. The presence of the redox couple peak at −0.2/−0.75 V can be attributed to the redox reactions of Mo_7_O_24_^6−^ ions (the anodic peak is slightly shifted towards the anodic potential compared to that of the (NH_4_)_2_Mo_7_O_24_ solution, see the insert in Figure 2a). The current density of the CV curves increases with the increase of precursor solution concentration. This allows one to predict that the thickness of MoS_2_ film will be increased in the order: solution 1.25 < solution 2.5 < solution 5.0.

### Morphology and structure of MoS_2_ thin films

**Figure 3:**
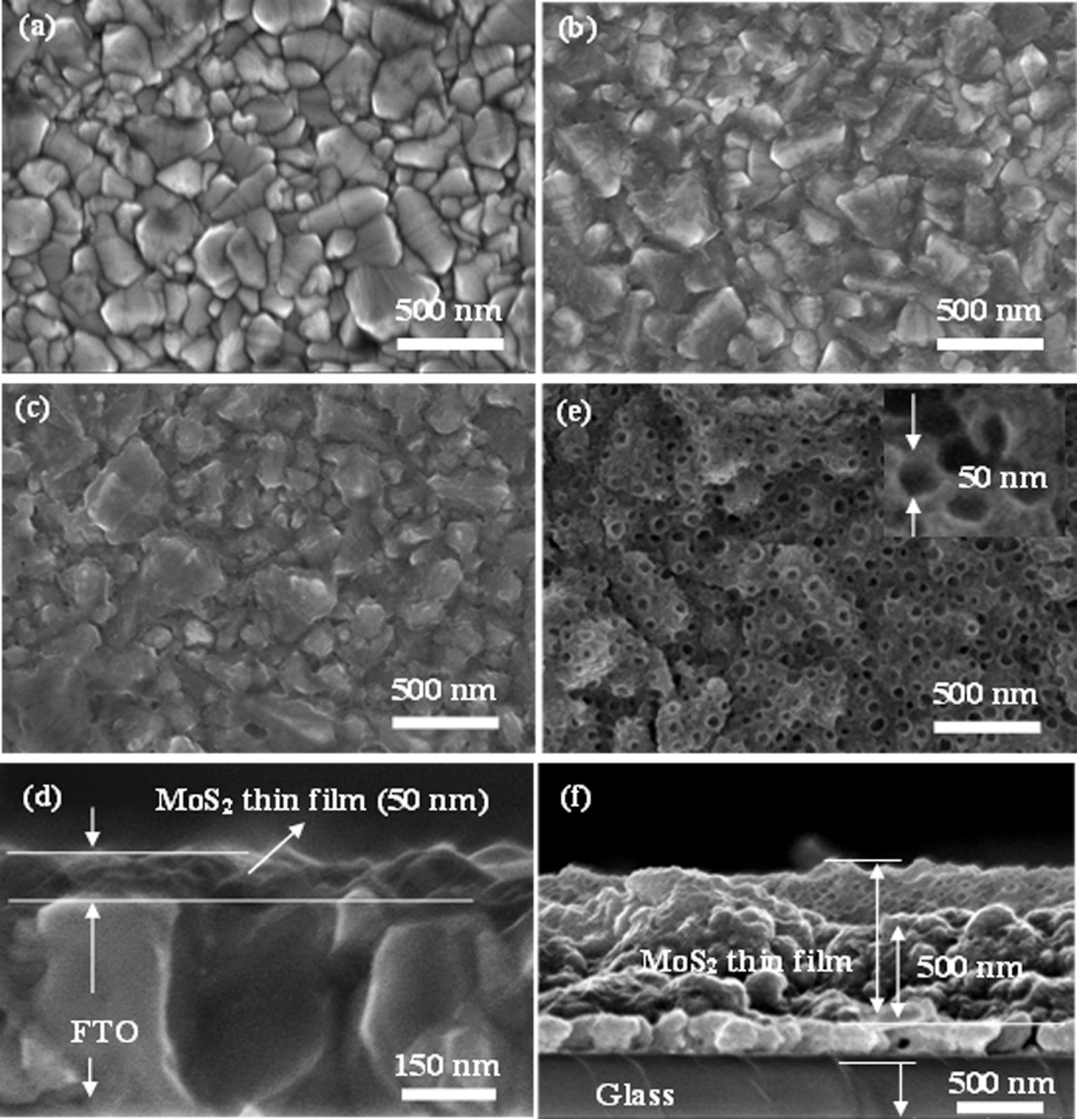
FE-SEM images (top view and cross-sectional view) of (a) FTO and (b–f) MoS_2_ deposited on FTO from different precursor solution concentrations: (b) solution 1.25, (c ,d) solution 2.5, and (e, f) solution 5.0.

Morphology and thickness of MoS_2_ films prepared on the FTO substrate were analyzed by FE-SEM. The MoS_2_ films formed from solutions 1.25 and 2.5 exhibited thin-layered structures, which exposed edge sites (Figure 3a–c). The same structure had been found in the reports of Falola and Lin [24,28]. However, the film thickness of MoS_2_ in these reports was thick compared to our results. Interestingly, the formation of MoS_2_ film from solution 5.0 showed a homogenous honeycomb-like structure (Figure 3e). The surface of the film consists of honeycomb grids with a diameter of around 50 nm (see Figure 3e, insert). The roughness of the films was further studied by AFM. The film with the honeycomb-like structure showed the highest average roughness (*S*_a_) and root mean square roughness (*S*_q_) of 24.179 and 30.443 nm, respectively (see Supporting Information File 1, Figure S1 and Table S1). To the best of our knowledge, this is the first report on this type of MoS_2_ film synthesized by CV. The potential range of the CV and the concentration of the precursor solution strongly affect the thickness and morphology of the MoS_2_ films. The thickness of MoS_2_ films was estimated from cross-sectional FE-SEM images. The formation of MoS_2_ from solutions 2.5 and 5.0 yielded thicknesses of about 50 nm and 500 nm, respectively (Figure 3d,f).

**Figure 4:**
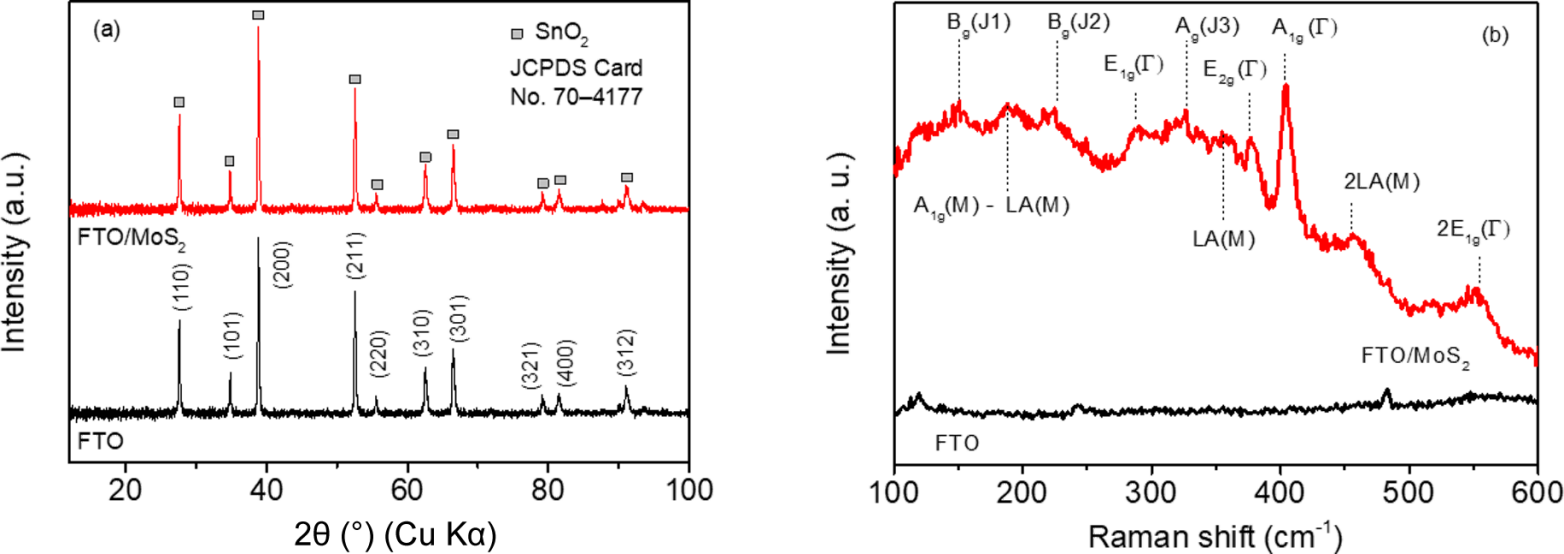
(a) XRD patterns and (b) Raman spectra of the FTO substrate and a thin film of MoS_2_ electrodeposited from solution 5.0.

The phase structure of the electrodeposited MoS_2_ thin films was identified by XRD and Raman analyses. The XRD pattern and the Raman spectrum of the MoS_2_ thin film deposited from solution 5.0 are presented in Figure 4. The XRD pattern of the MoS_2_/FTO samples shows only the peaks of the FTO substrate because the MoS_2_ thin film is amorphous or too thin (Figure 4a) [22–24]. Thus, the electrodeposited thin film was further characterized by Raman spectroscopy. The Raman spectrum of the MoS_2_/FTO sample showed the characteristic peaks of the 2H and 1T phases of MoS_2_ (Figure 4b). The appearance of the J_1_, J_2_, and J_3_ peaks around 150, 226, and 326 cm^−1^ confirmed the presence of the 1T metallic phase. Whereas the two Raman vibration modes, E_2g_ (in plane) and A_1g_ (out of plane), observed at 376 and 403 cm^−1^, respectively, are attributed to the 2H semiconductor phase [31–33]. The three first-order Raman modes, A_1g_, E_2g_, and E_1g_ (288 cm^–1^), are attributed to vibrational modes of the S–Mo–S layer. Other well-known multiphonon bands, namely A_1g_-LA (188 cm^−1^), LA (355 cm^−1^), 2LA (455 cm^−1^), and 2E_1g_ (553 cm^−1^) have also been observed [34–36]. The 1T phase exhibits a higher electroactivity than the 2H phase. Also, the 1T phase tends to transform into the 2H phase at high temperatures (Figure 4b) [1,17,36]. Therefore, the freshly prepared MoS_2_/FTO electrodes (without heat treatment) were used to examine the electrocatalytic activity towards the I_3_^−^/I^−^ redox couple as well as directly used as CEs for DSSCs.

### Electroactivity of MoS_2_ CEs

**Figure 5:**
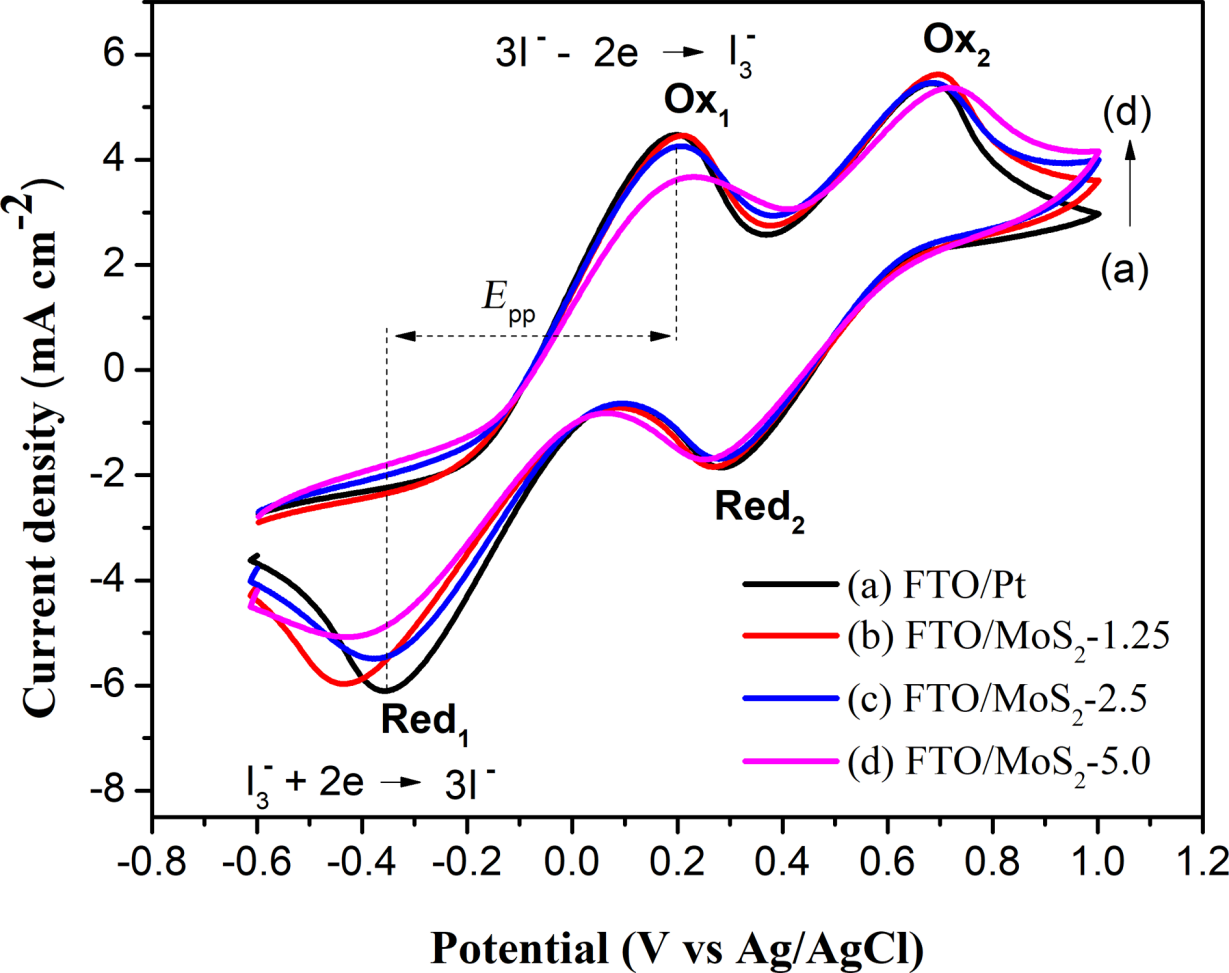
CV curves of MoS_2_ CEs prepared with different concentrations of reaction precursors compared to that of Pt CE, recorded in ACN solution of 10 mM I_2_, 20 mM KI, and 0.1 M LiClO_4_; a scan rate of 100 mV·s^−1^ was used.

The electrocatalytic activity of MoS_2_ CEs towards the I_3_^−^/I^−^ redox couple was investigated and compared to that of a Pt CE. As can be seen in Figure 5, there are two redox pairs (Ox_1_/Red_1_) and (Ox_2_/Red_2_). These redox peaks were well defined as the oxidation and reduction of iodide and triiodide (3I^−^ − 2e^−^ → I_3_^−^ (Ox_1_), I_3_^−^ + 2e^−^ → 3I^−^ (Red_1_) and 2I_3_^−^ − 2e^−^ → 3I_2_ (Ox_2_), 3I_2_ + 2e^−^ → 2I_3_^−^ (Red_2_) [6–9].

Since the reduction of I_3_^−^ to I^−^ on the CE plays a vital role in the regeneration of the oxidized dye molecules on the photoanode of the DSSCs, the electrocatalytic behavior of MoS_2_ CEs was further evaluated regarding the first redox couple (Ox_1_/Red_1_). Various parameters including anode/cathode peak potentials (*E*_pOx1_, *E*_pRed1_), peak-to-peak voltage separation (*E*_pp_), and anode/cathode peak current densities (*J*_Ox1_, J_Red1_) were calculated and presented in Table 1. The *E*_pp_ value for MoS_2_ CEs was slightly larger than that of Pt CE confirming their excellent electrocatalytic activity. The *J*_Red1_ values of MoS_2_ CEs decreased in the order: MoS_2_-1.25 ≈ Pt > MoS_2_-2.5 > MoS_2_-5.0 (see Table 1). This demonstrated that increasing the concentration of the precursor solution resulted in the increase in the thickness of the MoS_2_ film and, thus, reduced the electrocatalytic activity.

**Table 1:** Electrochemical parameters from CV measurements of MoS_2_ and Pt CEs.

CE	*E*_pOx1_ (V)	*E*_pRed1_ (V)	*E*_pp_ (V)	*J*_Ox1_ (mA·cm^−2^)	*J*_Red1_ (mA·cm^−2^)

Pt	0.195	−0.355	0.550	4.450	−6.059
MoS_2_-1.25	0.208	−0.432	0.640	4.476	−5.965
MoS_2_-2.5	0.208	−0.373	0.581	4.257	−5.511
MoS_2_-5.0	0.226	−0.415	0.641	3.695	−5.057

**Figure 6:**
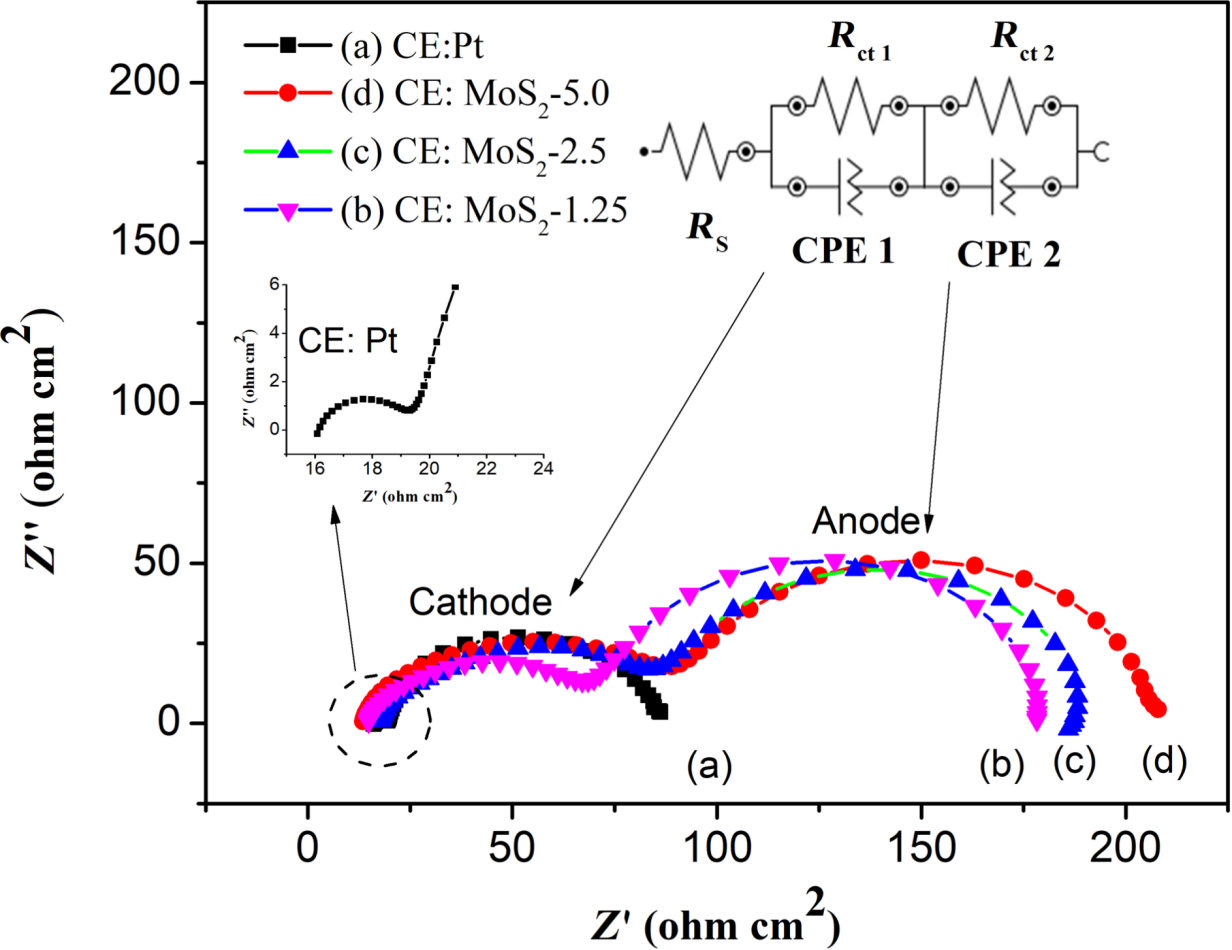
Nyquist plots of DSSCs using different MoS_2_/FTO and Pt/FTO CEs, the inset shows the equivalent circuit model.

Further study of the electrocatalytic behavior of the MoS_2_ CEs was carried out by using the EIS technique under dark conditions using full cells assembled from different MoS_2_/FTO CEs or a Pt/FTO CE. The Nyquist plots for these cells exhibited two semicircles as presented in Figure 6. The first semicircle in the high-frequency region is associated with the reduction of I_3_^−^ at the cathode (CE/electrolyte), while the second one in the low-frequency region is attributed to electron transport in the TiO_2_ film in the back reaction at the TiO_2_/electrolyte interface (TiO_2_/dye/electrolyte). EIS data were fitted using an equivalent-circuit model including the series resistances of electrolyte and FTO substrate (*R*_s_) and the charge-transfer resistances on the CE/electrolyte and TiO_2_/dye/electrolyte interfaces (*R*_ct1_ and *R*_ct2_) associated with the corresponding constant phase elements (CPE1 and CPE2) as described in Figure 6 (Figure 6, insert).

The fit values for *R*_s_ and *R*_ct1_ reflect the catalytic behavior of CEs in DSSCs and are presented in Table 2. The *R*_s_ value for MoS_2_ CE-based DSSCs slightly increased with the thickness of the films and is comparable to that of Pt CE-based DSSCs. This is due to the high conductivity of the metallic 1T phase of MoS_2_ and is in good agreement with Raman analysis. Under dark conditions, the *R*_ct1_ value for MoS_2_ CE-based DSSCs (from 52.6 to 78.5 Ω·cm^2^) was found to be significantly higher than that of Pt CE-based DSSCs (3.6 Ω·cm^2^), indicating slower charge transfer kinetics at the MoS_2_/electrolyte interface compared to the Pt/electrolyte interface. The high peak current density value for I_3_^−^ reduction from CV analysis obtained for MoS_2_-based CEs was therefore attributed to the high number of catalytically active sites located on the edge planes of the MoS_2_ films.

**Table 2:** Photovoltaic parameters and EIS data of the DSSCs based on different MoS_2_ CEs and a Pt CE.

CE	*J*_sc_ (mA·cm^−2^)	*V*_oc_ (V)	FF	η (%)	*R*_s_ (Ω·cm^2^)	*R*_ct1_ (Ω·cm^2^)	τ (ms)

Pt	15.30	0.75	0.75	8.66	16.1	3.60	23
MoS_2_-1.25	16.30	0.69	0.66	7.33	14.8	52.6	30
MoS_2_-2.5	14.85	0.68	0.63	6.39	16.6	65.8	31
MoS_2_-5.0	14.90	0.67	0.53	5.31	17.5	78.5	40

### DSSC performance

**Figure 7:**
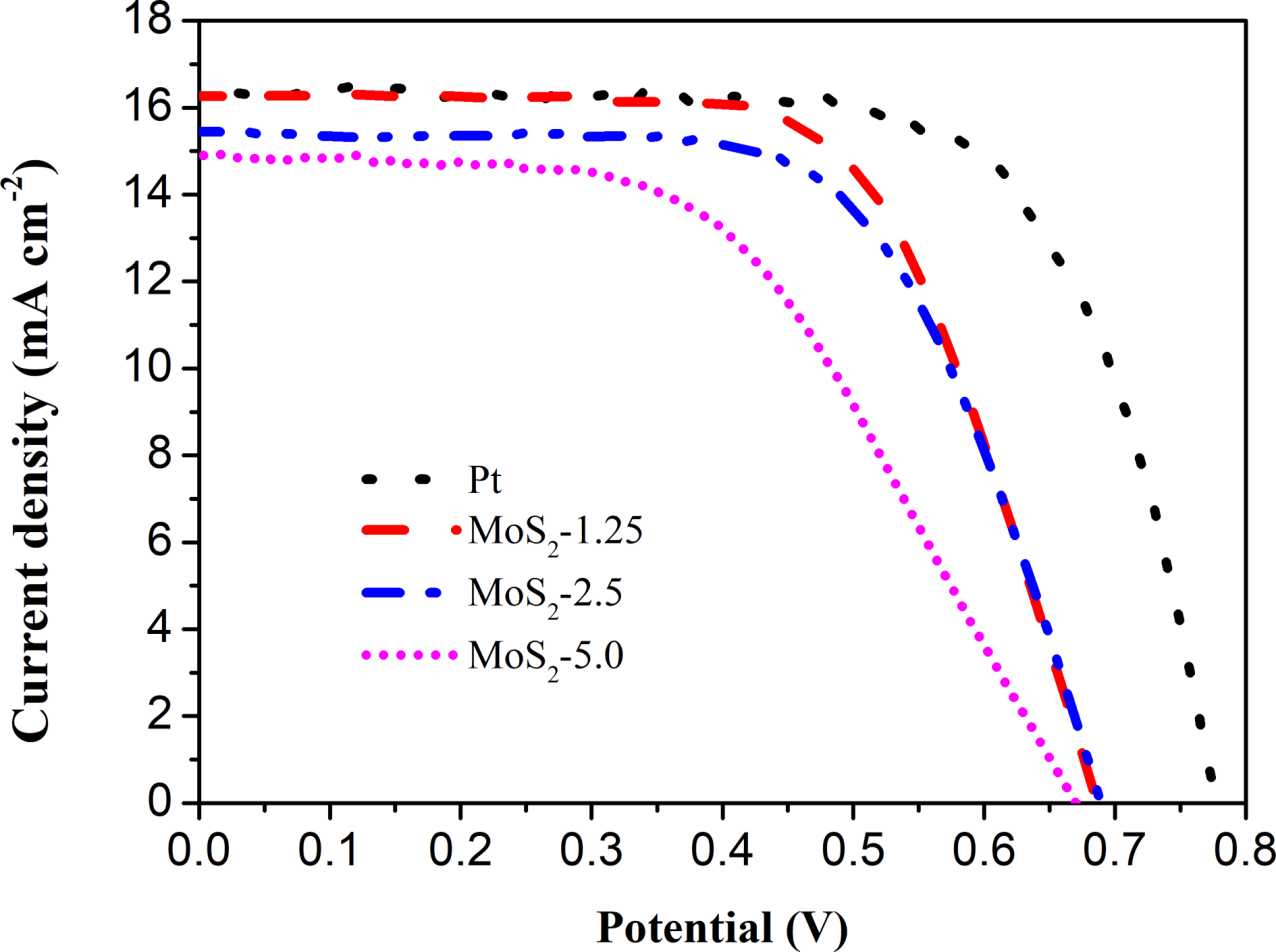
Photovoltaic performance of DSSCs fabricated with different MoS_2_/FTO and Pt/FTO CEs.

To further evaluate the effect of MoS_2_ morphology and thickness on the performance of the DSSCs, the photovoltaic performance of DSSCs using different MoS_2_ CEs was investigated under illumination. The *J*–*V* curves and the corresponding photovoltaic parameters of DSSCs are given in Figure 7 and Table 2, respectively. The DSSC using MoS_2_-1.25/FTO CE displayed an excellent photovoltaic performance compared to that with a Pt/FTO a CE. In particular, the obtained value of 16.3 mA·cm^−2^ for the short-circuit photocurrent (*J*_sc_) was found to be higher than that of Pt/FTO CE (15.3 mA·cm^−2^). This is attributed to the high number of catalytically active sites together with the low resistance of this MoS_2_ film.

Although the electrocatalytic ability regarding the reduction of I_3_^−^ of MoS_2_ was lower than that of Pt, other parameters including an open-circuit voltage (*V*_oc_) of 0.69 V, a fill factor (FF) of 0.66, and a PCE of 7.33% of this MoS_2_ CE-based DSSCs were comparable to those of a DSSC based on a Pt CE (*V*_OC_ = 0.75 V, FF = 0.75, PEC = 8.66%) and to values found in other reports (Table 3). The PCE for DSSCs using MoS_2_ with different thicknesses decreased in the order: MoS_2_-1.25 (very thin film) > MoS_2_-2.5 (50 nm) > MoS_2_-5.0 (500 nm). This is in good agreement with the trend of *R*_s_ and *R*_ct1_ values (see Table 2 and Table 3). The effect of film thickness on the electrical conductivity of the MoS_2_ films was also investigated by *I*–*V* measurements (see Supporting Information File 1, Figure S2 and Table S1). The electrical conductivity of the as-prepared MoS_2_ decreased in the order: MoS_2_-1.25 (75 mS·cm^−1^) > MoS_2_-2.5 (61 mS·cm^−1^) > MoS_2_-5.0 (46 mS·cm^−1^). This suggests that the thickness of the MoS_2_ film has a significant effect on the catalytic ability and photovoltaic performance of the CE in DSSCs. Additionally, the electron lifetime (τ) indicates the recombination kinetics of electrons in the mesoscopic TiO_2_ film of the DSSCs. This parameter can be calculated from the peak frequency (*f*_max_) of the low-frequency semicircles (τ = 1/2π*f*_max_). The τ values measured under dark conditions for DSSCs devices fabricated from various CEs are presented in Table 2. The τ values for DSSCs fabricated with MoS_2_ CEs were higher than those of a DSSC using a Pt CE and increased in the order: DSSCs-MoS_2_-1.25 < DSSCs-MoS_2_-2.5 < DSSCs-MoS_2_-5.0. A longer electron lifetime indicates a slower recombination process within the DSSCs fabricated with MoS_2_/FTO CEs. The stability of the devices was tested by repeating the *I*–*V* measurements every week (Supporting Information File 1, Figure S3). The PCE value of a DSSC fabricated using MoS_2_-1.25 showed a slight decrease of 2.6% and reached stability after two weeks of testing under ambient conditions. This suggested that the MoS_2_-1.25/FTO CE has reversible redox activity and electrochemical stability. The electrochemical stability of the MoS_2_/FTO CE should provide long-term stability for solar cell devices. However, more work needs to be done to improve the efficiency of this DSSC device [36–39].

**Table 3:** Performance summary of MoS_2_-based CEs for DSSCs.

MoS_2_ CE	Method	*V*_oc_ (V)	*J*_sc_ (mA·cm^−2^)	FF	η (%)	Ref

MoS_2_/FTO	chemical bath deposition	0.73	15.92	0.61	7.14	[1]
MoS_2_/FTO	sputtering	0.71	13.17	0.64	6.00	[9]
MoS_2_/FTO	hydrothermal	0.70	18.37	0.58	7.41	[10]
1T MoS_2_/FTO	hydrothermal	0.73	18.76	0.52	7.08	[11]
2H MoS_2_/FTO	hydrothermal	0.73	6.78	0.35	1.72	[11]
MoS_2_/FTO	hydrothermal	0.74	16.96	0.66	8.28	[12]
porous MoS_2_/FTO	hydrothermal/spin coating	0.76	15.4	0.53	6.35	[13]
flower-shaped MoS_2_/FTO	hydrothermal/spin coating	0.70	13.73	0.52	5.23	[13]
MoS_2_/FTO	wet-chemical process	0.68	18.46	0.58	7.01	[14]
MoS_2_/FTO	spin coating/thermal reduction	0.73	16.91	0.52	6.35	[15]
multilayered MoS_2_/FTO	spray coating	0.75	15.81	0.25	2.92	[16]
few-layered MoS_2_/FTO	exfoliation of ML-MoS_2_ powder and spray coating technique	0.74	14.90	0.16	1.74	[16]
MoS_2_ nanoparticles/FTO	thermal decomposition	0.75	14.72	0.49	5.41	[16]
MoS_2_/FTO	potentiostatic	0.72	15.68	0.63	7.16	[22]
MoS_2_/FTO	potentiostatic	0.78	16.18	0.54	6.89	[24]
MoS_2_/FTO	potential reversal	0.76	16.16	0.71	8.77	[24]
layered MoS_2_/FTO	cyclic voltammetry	0.69	16.29	0.66	7.33	this work
honeycomb-like MoS_2_/FTO	cyclic voltammetry	0.67	14.90	0.53	5.31	this work

## Conclusion

We synthesized successfully MoS_2_ thin films with layered or honeycomb-like structures onto FTO substrates by the CV route. The morphology and thickness of the MoS_2_ films can be effectively controlled by adjusting the concentration of the precursor solution. In detail, MoS_2_ formed a layered thin film with a thickness of about 50 nm when the concentration level ratio between (NH_4_)_2_Mo_7_O_24_ and Na_2_S was 2.5/15 (solution 2.5, see Experimental section). Honeycomb MoS_2_ was formed with a thickness of about 500 nm from solution 5.0 (with a concentration level ratio between (NH_4_)_2_Mo_7_O_24_ and Na_2_S of 5/30). In addition, as-prepared MoS_2_ films have been used as an alternative CE to Pt in DSSCs. The short-circuit photocurrent (*J*_sc_) was higher than that of a Pt/FTO CE. Moreover, the highest performance of solar cells was found with the layered MoS_2_ film thank to good electrical conductivity, a high number of catalytically active sites, and the thickness of the MoS_2_ film. The MoS_2_/FTO films could be applied as non-Pt electrodes for DSSCs in the near future.

## Experimental

### Materials and reagents

Ammonium molybdate tetrahydrate ((NH_4_)_6_Mo_7_O_24_·4H_2_O, 99.98%), sodium sulfide nonahydrate (Na_2_S·9H_2_O, 99.99%), potassium chloride (KCl, 99%), acetonitrile (ACN, CH_3_CN, 99%), dimethyl sulfoxide (DMSO, (CH_3_)_2_SO, 99.5%), ethanol (EtOH, CH_3_CH_2_OH, 99.8%), guanidinium thiocyanate (NH_2_C(=NH)NH_2_·HSCN, 99%), iodine (I_2_, 99.8%), 1-methyl-3-propylimidazolium iodide (C_7_H_13_IN_2_, 98%), 4-*tert*-butylpyridine (C_9_H_13_N, 98%), valeronitrile (CH_3_(CH_2_)_3_CN, 99.5%), chloroplatinic acid hexahydrate (H_2_PtCl_6_·6H_2_O, ≥37.50% Pt), sulfuric acid (H_2_SO_4_, 95–98%), polyvinylpyrrolidone (PVP, (C_6_H_9_NO)*_n_*, average *M*_w_ 10,000), and sodium borohydride (NaBH_4_, 99%) were purchased from Sigma-Aldrich (Germany). Low-temperature thermoplastic sealant (Surlyn, 25 μm), 18NR-T transparent titania paste (particle size of 20 nm), 18NR-AO active opaque titania paste (particle sizes of 20 and 450 nm), fluorine-doped tin oxide (FTO, TEC8 glass plates, 8 Ω·cm^−2^, 2.2 mm thickness), and N719 industry standard dye (N719) were purchased from Dyesol (Australia). All commercial chemicals were of analytical grade and were used as supplied without further purification.

### Electrodeposition of MoS_2_ thin films

Thin films of MoS_2_ with different morphologies were electrodeposited onto FTO substrates by the CV method. MoS_2_ electrodeposition was carried out using an Autolab 302 N (Eco chemie, Netherlands) connected to a three-electrode cell. Accordingly, a Pt mesh, an Ag/AgCl (ALS, Japan), and the FTO plate (1.5 × 1.5 cm) were used as the counter electrode (CE), the reference electrode (RE), and the working electrode (WE), respectively. Prior to CV electrodeposition, the FTO substrates were first cleaned in a 1% Hellmanex solution at 70 °C for 30 min in an ultrasonic bath, then washed three times in distilled water, dried by nitrogen flow, and finally treated in a UV ozone chamber for 5 min to obtain the cleaned FTO electrode. Precursor solutions were prepared by dissolving (NH_4_)_6_Mo_7_O_24_ (*x* mM) and Na_2_S (*y* g·L^−1^) in distilled water, the pH of the solution was adjusted to 6.0 using a 20% (v/v) H_2_SO_4_ solution, KCl (0.1 M) was used as the supporting electrolyte. The concentration ratio between (NH_4_)_2_Mo_7_O_24_ and Na_2_S was kept constant (*x*/*y* = 1:6) with different concentration levels including 1.25:7.5 (solution 1.25), 2.5:15 (solution 2.5), and 5:30 (solution 5.0). The CV measurement was performed under a dynamic potential between −1.0 V and 1.0 V for ten cycles at a scan rate of 100 mV·s^−1^ in a nitrogen atmosphere.

### Fabrication of DSSCs

DSSCs with an active area of 0.25 cm^2^ were assembled using the pre-cleaned FTO plates (1.5 × 1.5 cm) for the fabrication of anode and cathode. For cathode preparation, MoS_2_/FTO CEs were prepared with different morphologies from the above MoS_2_ samples. The obtained CEs were designated as MoS_2_-1.25/FTO, MoS_2_-2.5/FTO, and MoS_2_-5.0/FTO. For comparison, a Pt-based CE (Pt/FTO) was fabricated by soaking the cleaned FTO glasses in PVP–platinum suspension at 45 °C for 5 min, followed by washing with distilled water. The PVP–platinum suspension was prepared as follows: First, 1.0 g of H_2_PtCl_6_ was dissolved in 150 mL of distilled water, then 0.5 g PVP was added to the above solution under stirring for 10 min. Finally, to this solution, NaBH_4_ solution (1.17 g NaBH_4_ was dissolved in 124.8 mL of distilled water, stirred for 3 min) was added at a rate of 1.5 mL/min until the color of the mixture solution turned into black.

For the fabrication of the photoanodes, the cleaned FTO electrodes were first pretreated by immersion in a 40 mM TiCl_4_ solution at 70 °C for 30 min and rinsed with distilled water and ethanol. The treated FTO electrodes were then successively coated with a transparent 18NR-T titania paste (three layers) and an active opaque 18NR-AO titania paste (one outer layer) by the screen-printing method using 43T mesh. The printed electrodes were dried at room temperature for 5 min, then at 120 °C for 5 min after each printed layer, and finally heated at 450 °C under airflow for 30 min. When the temperature was cooled down to 70 °C, the electrodes were dipped in N719 dye (0.3 M N719 in the mixture DMSO/EtOH, v/v = 1:9) for 12 h to obtain the photoanodes.

For cell assembly, the DSSCs were fabricated in a nitrogen atmosphere using a glove box. Typically, the photoanode and the MoS_2_-based CE were sealed together by a hot-melt Surlyn film using a thermopress, pressed at 170 °C for 15 s. The electrolyte solution (0.1 M guanidinium thiocyanate, 0.03 M iodine, 1 M 1-methyl-3-propylimidazolium iodide, 0.5 M 4-*tert*-butylpyridine in a mixture of valeronitrile/acetonitrile with a volume ratio of 0.15:0.85) was then injected into the cell through predrilled holes on the CE. The holes were then covered with a thin glass slide using the same thermopress method as described above to obtain DSSC devices. DSSCs assembled using the CE fabricated from precursor solutions 1.25, 2.5, and 5.0 were denoted as DSSCs-MoS_2_-1.25, DSSCs- MoS_2_-2.5, and DSSCs-MoS_2_-5.0, respectively. For comparison, DSSCs based on Pt/FTO CE (DSSCs-Pt) were also fabricated under the same conditions.

### Characterizations of MoS_2_ thin films

X-ray diffraction (XRD) analysis was carried out using a D8 Advance (Bruker, Germany) with a copper anode (λ_Kα_ = 1.54 Å). Raman spectroscopy measurements were performed on a LabRAM HR 800 Raman Spectrometer (HORIBA Jobin Yvon) with an excitation laser source at 532 nm. The morphology of MoS_2_ thin films was analyzed by an ultrahigh-resolution field-emission scanning electron microscope (FE-SEM, Hitachi SU-8010, Japan). The electrochemical catalytic activity of the MoS_2_-based CEs was studied regarding the I_3_^−^/I^−^ redox couple (prepared with 10 mM I_2_, 20 mM KI, and 0.1 M LiClO_4_ in acetonitrile) and compared to that of a FTO/Pt CE by CV.

### DSSC characterizations

Photoelectrochemical measurements were performed using an Oriel Sol1A class ABB solar simulator (Oriel-Newport-USA, Model No. 94061A). Simulated sunlight of 100 mW·cm^−2^ (1 sun) was generated and corrected by a 1000 W Xe lamp and an AM 1.5 filter. The photocurrent density–voltage (*J*–*V*) curves of the DSSCs were measured using a Keithley model 2400 multisource meter. Electrochemical impedance spectroscopy (EIS) of the fabricated DSSCs was carried out using an Autolab 302 N equipped with a FRA 32M module. The EIS measurements were carried out at open-circuit voltage with an alternating voltage amplitude of 10 mV under dark conditions in a frequency range between 0.01 Hz and 100 kHz. The efficiency of the DSSCs was analyzed and reported as the average of three cells.

## Supporting Information

File 1 Additional experimental data.
