# Supplementary material for: Influence of thickness and morphology of MoS2 on the performance of counter electrodes in dye-sensitized solar cells
Source: Beilstein J Nanotechnol. 2022 Jun 17;13:528–37. doi: 10.3762/bjnano.13.44 (PMC9235830; doi:10.3762/bjnano.13.44)
Supplement: File 1 — Additional experimental data. [file Beilstein_J_Nanotechnol-13-528-s001.pdf]

BEILSTEIN JOURNAL OF NANOTECHNOLOGY

Supporting Information

for

Influence of thickness and morphology of MoS_2 on the performance of counter electrodes in dye-sensitized solar cells

Lam Thuy Thi Mai, Hai Viet Le, Ngan Kim Thi Nguyen, Van La Tran Pham, Thu Anh Thi Nguyen, Nguyen Thanh Le Huynh and Hoang Thai Nguyen

Beilstein J. Nanotechnol. **2022,** *13,* 528–537. doi:10.3762/bjnano.13.44

Additional experimental data

License and Terms: This is a supporting information file under the terms of the Creative Commons Attribution License (https://creativecommons.org/licenses/by/4.0). Please note that the reuse, redistribution and reproduction in particular requires that the author(s) and source are credited and that individual graphics may be subject to special legal provisions.
The license is subject to the *Beilstein Journal of Nanotechnology* terms and conditions: (https://www.beilstein-journals.org/bjnano/terms)

AFM and electrical conductivity characterization of devices

The AFM method was performed using a FlexAFM (Nanosurf, Switzerland) under ambient conditions using a cantilever type NCLR. The Electrical conductivity of the MoS_2 films deposited on the FTO substrates was measured with a Keithley model 2400 multisource meter at room temperature.

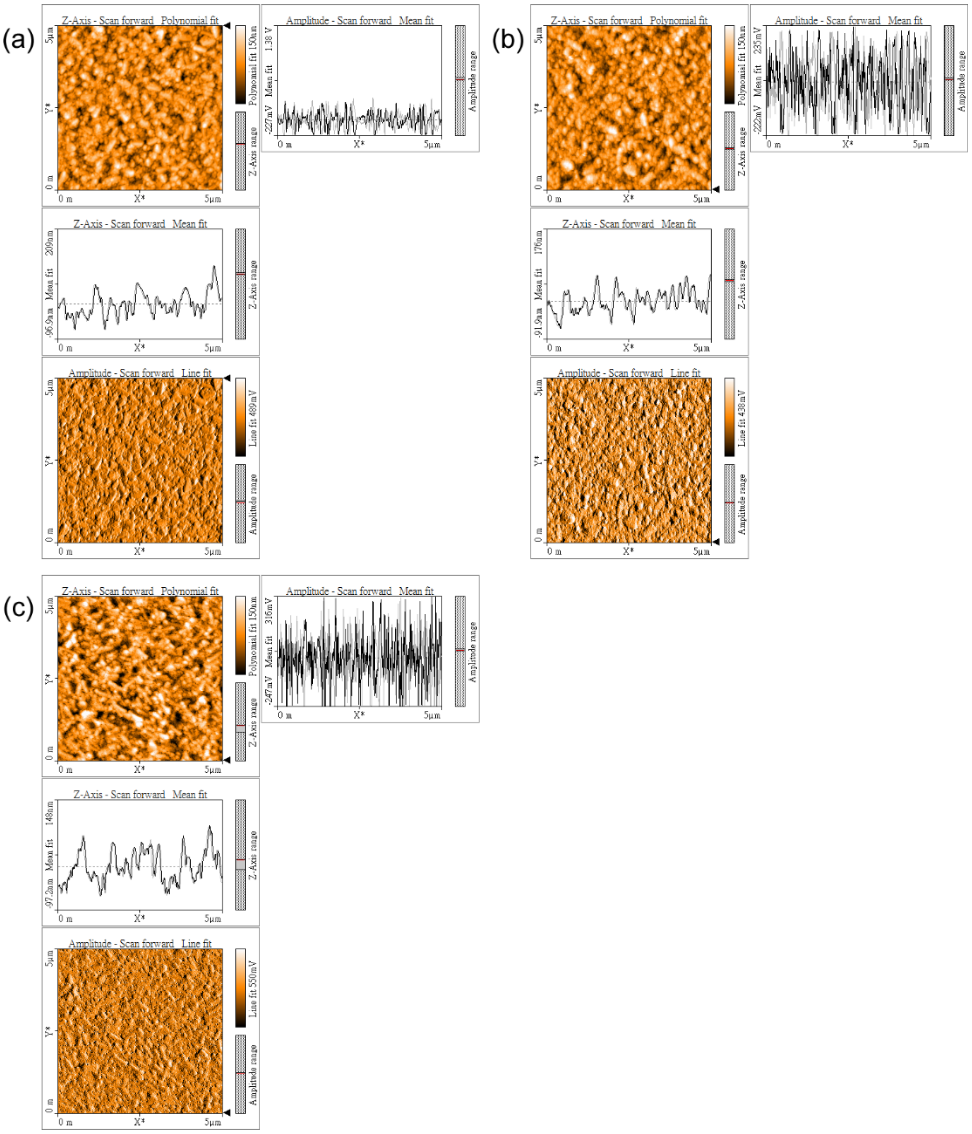

Figure S1: AFM images of (a) MoS_2-1.25/FTO, (b) MoS_2-2.5/FTO, and (c) MoS_2-5.0/FTO.

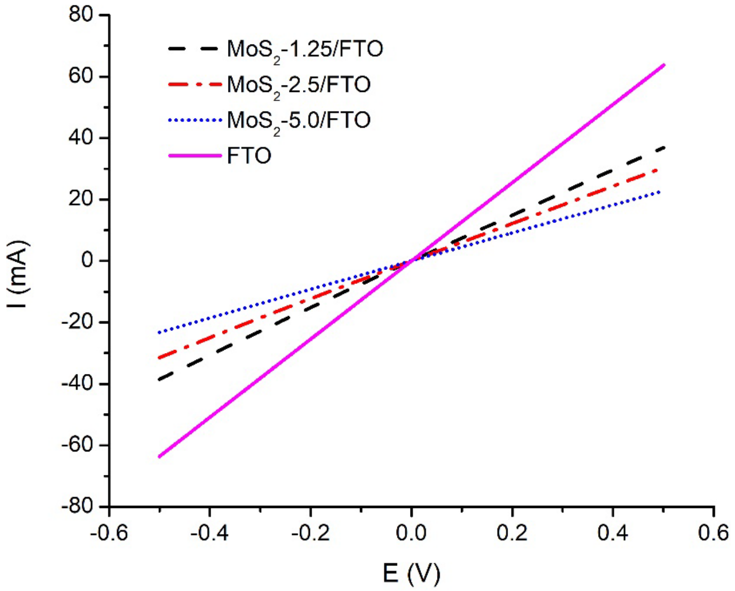

Figure S2: Current–voltage (*I*–*V*) plots of MoS_2 thin films compared to that of the FTO substrate.

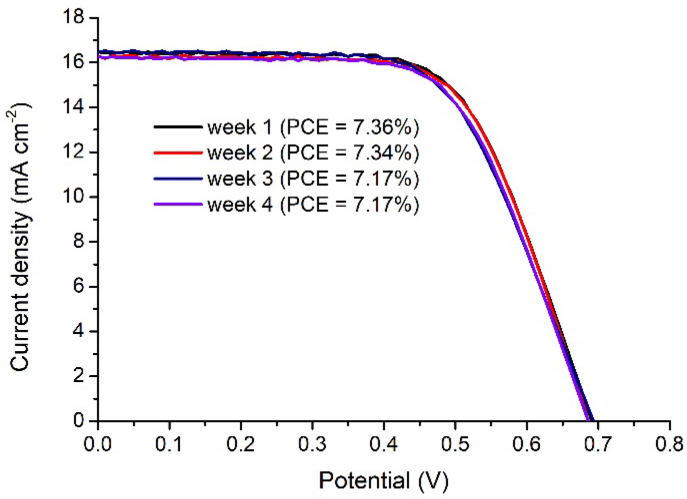

Figure S3: Photovoltaic performance of the DSSCs fabricated from MoS_2-1.25/FTO over four weeks of testing.

Table S1: AFM image roughness and *I–V* electrical conductivity of the MoS_2 thin films.

Sample	AFM-images roughness		*I–V* electrical conductivity ($mS \cdot cm^{-1}$)
	average roughness (S_a, nm)	root mean square roughness (S_q, nm)	
MoS_2-1.25	19.111	23.895	75
MoS_2-2.5	19.822	24.981	61
MoS_2-5.0	24.179	30.443	46
